# Supplementary material for: The psychology of professional and student actors: Creativity, personality, and motivation
Source: PLoS One. 2020 Oct 22;15(10):e0240728. doi: 10.1371/journal.pone.0240728 (PMC7580901; doi:10.1371/journal.pone.0240728)
Supplement: Attachment — Submitted filename: renamed_594c2.docx [file pone.0240728.s001.docx]

PlosOne Review – March 15, 2020

Manuscript ID - PONE-D-20-02455

Title: The Psychology of Professional and Student Actors: Creativity, Personality, and Motivation

**Main Findings and Significance to the Discipline**

The main findings of this study include personality traits, divergent thinking responses, creative activities, emotional intelligence and grit. The study demonstrated factors that differentiate professional actors, from student actors and non-actors. The statistical analyses were novel and strengthened the reporting of the findings. Because actors are under-researched, this study is important and necessary.

**General Comments:**

In general, the manuscript is very well written and clearly organized. The authors have carefully outlined the study protocol and methods of analysis. They have situated their findings within the larger field of research and their findings are important. The authors have included many important references that support the purpose of their study and the importance of their findings. They have also provided enough information so that the study can be replicated and provided information about how to access their original data. It is recommended that the authors also include work by Zorana Ivcevic and colleagues as well as Thalia Goldstein and Ellen Winner. They have all conducted work on emotional intelligence, theory of mind, and empathy in actors and their relationship to creativity. Lastly, a careful edit will catch inconsistencies in citation formatting and reference formatting.

**Abstract:**

The abstract is well organized and concise. It would be helpful to include the number of actors, student actors and non-actors in the abstract as well.

**Review of Literature:**

This section is generally well organized. Providing an historical overview positions the current study and highlights the importance of this study. In the discussion on empathy the authors should include research findings by Winner and Goldstein. They also examined empathy and theory of mind in actors.

**Methods:**

This section is detailed and situates this study so that it could be easily replicated. Can the authors provide more information on the control sample? The control group also clearly engages in creative activities given their responses. The measures and the statistical analyses were detailed.

**Results:**

In combination with the tables and figure, the results were fully explained. The authors also offer solid reasons for their statistical choices. The summary results for each measure was very helpful.

**Discussion and Conclusions:**

The overall findings were summarized succinctly. The limitations were relevant. As stated by the authors, longitudinal work is needed to understand the large attrition rate as student actors move into a professional career. It would be helpful to provide a stronger statement differentiating student actors from professional actors, especially because student actors who are trained in a conservatory setting are working in stable environments, hence their willingness to take risks would be higher. Age further influences some of these differences, a factor that was minimally addressed in this study.

**References:**

A careful edit is required to correct inconsistencies in formatting.

**Tables and Figures:**

The tables and figure are helpful and clearly outlined.

**Specific Comments:**

P. 3 – change (Kogan, 2002) to the correct formatting.

P. 5 – Jague – should be spelled Jaque to match the reference listing

P. 12 – change doctoral agree to doctoral degree

p. 15 – Likert is incorrectly spelled

p. 31 – there is a double wording of “that suggests that suggests”
