# Supplementary material for: The psychology of professional and student actors: Creativity, personality, and motivation
Source: PLoS One. 2020 Oct 22;15(10):e0240728. doi: 10.1371/journal.pone.0240728 (PMC7580901; doi:10.1371/journal.pone.0240728)
Supplement: Attachment — Submitted filename: manuscript review- May 2020 The Psychology of Professional and Student Actors.docx [file pone.0240728.s002.docx]

“The Psychology of Professional and Student Actors: Creativity, Personality, and Motivation”

**Revise and Resubmit**

The manuscript, *The Psychology of Professional and Student Actors: Creativity, Personality, and Motivation*, is an interesting paper describing a newer approach to identifying group membership among actors and non-actors using a novel set of measures of creativity and other psychological dimensions. Although it has many strengths, including the LASSO approach, there are some suggestions that the authors might address to strengthen the paper.

Major revisions

1. The “summary of extant work” section of the introduction could be streamlined. For instance, it begins as if the research is presented chronologically (e.g., “earliest work”), but then goes into negative psychological attributes, cognitive and expertise development, followed by a final paragraph that overlaps with prior content. I think taking the final paragraph in that section and incorporating it into other sections of the paper would improve flow (e.g., paragraph of Nettle’s personality and ASD components could be incorporated into the first paragraph of section or the paragraph on negative emotional attributes. Also, worth possibly including is the recent Gentzler, DeLong, and Smart (2019) for their comparisons of student acting majors and non-actors on a variety of psychological dimensions. For instance, they assessed temperament which can be regarded as similar to personality.
2. In the divergent thinking assessment section, I thought the points made by the authors as to why that measure could be a useful indicator of performance for actors was good. However, I think there is much to be elaborated. First, has that measure actually been assessed among actors? It’s lack of use for predictive power for determining group membership is made clear, but if it has been assessed as an outcome among participants is unclear as written.
3. More detail about the personality facets could be included. It’s unclear if the authors’ comments about those that load onto the Big 5 is in reference to the emotional intelligence and grit previously mentioned, or if they are referring to other facets. Given that the Big5 dimensions are often broken down to the facet-level, more clarity is warranted.
4. A clear statement about the main benefits of the project is warranted in the introduction. The first time the authors describe the benefits concretely, is in the final paragraph of the paper. The authors begin the manuscript with noting the research questions that stem from comparing the unique lifestyles of actors to non-actors, but it’s unstated as to why that matters. Although I agree it’s a great research question quite interesting to study, briefly mentioning the benefits of a study like this would greatly strengthen the selling point of the paper.
5. Regarding the undergraduate acting majors, what year in the program were they? Do you have any indicators of how much experience those students had on stage (e.g., number of productions performed in)? I would think that those who participated in more productions would be better actors compared to less experienced students and may have important implications for the other qualities measured. Also, was there an index to confirm the non-acting participants had not performed in any productions before?
   a. Also, it would help to know if there was any difference in the students who might be “theatre” vs “acting” students. For example, could there be students with different training/ coursework (e.g., vocal musical training for musical theatre vs. vocal training for speech alone), creating subgroups within the major? If there are differences, it would be a nice addition to know if there are any differences in the students’ trainings and experience and any implications of that. If there are no differences and “theatre and acting” is really the same program at U of Denver, it would be useful to simply adjust the wording in the paper to indicate that’s just what the program is called.
6. I thought that the description of the LASSO technique in the results section was thorough and easy to follow. I found it interesting that some variables that showed no univariate differences between groups were selected for the LASSO model predicting actor status. I think some elaboration is needed to explain why that happened, and its implications in future research for statistical methods and for understanding actors from non-actors.
7. For some findings, descriptive implications are missing from the results or discussion (e.g., emotional intelligence measures, tolerance of uncertainty). For instance, a description of implication regarding the unexpected finding at the univariate level for emotional intelligence and the more anticipated finding with LASSO is warranted. Additionally, the implications regarding findings with tolerance of uncertainty are not elaborate. For example, the finding that students are more tolerant of uncertainty than professionals is not surprising to me given that students are less likely to be solely responsible for their own livelihood (e.g., receiving financial aid or assistance from caregivers while in college). Adding brief explanations for why the separate dimensions of emotional intelligence, personality, etc. were found would be very useful.

Minor revisions

1. A few typos are apparent (e.g., missing punctuation after citation brackets, missing “s” for the plural form of the word “responses”, spelling of “facets” as “facts”).
