# Supplementary material for: The psychology of professional and student actors: Creativity, personality, and motivation
Source: PLoS One. 2020 Oct 22;15(10):e0240728. doi: 10.1371/journal.pone.0240728 (PMC7580901; doi:10.1371/journal.pone.0240728)
Supplement: Attachment — Submitted filename: Revision_Review for Plus One.docx [file pone.0240728.s003.docx]

Revision: Manuscript Number: PONE-D-20-02455R1

Title: The Psychology of Professional and Student Actors: Creativity, Personality, and Motivation

Reviewer Response – June 24, 2020

The authors have addressed all concerns raised and the revision has made this a very strong paper. The authors provided substantial details to explain the revisions and they have clearly given this revised manuscript substantial thought and careful consideration. This is an important study that addresses an under-researched population.
