# Supplementary material for: The psychology of professional and student actors: Creativity, personality, and motivation
Source: PLoS One. 2020 Oct 22;15(10):e0240728. doi: 10.1371/journal.pone.0240728 (PMC7580901; doi:10.1371/journal.pone.0240728)
Supplement: Attachment — Submitted filename: manuscript- May 2020 The Psychology of Professional and Student Actors- RE-REVIEW.docx [file pone.0240728.s004.docx]

“The Psychology of Professional and Student Actors: Creativity, Personality, and Motivation”

**Accept with Revisions**

The manuscript, *The Psychology of Professional and Student Actors: Creativity, Personality, and Motivation*, is an interesting paper describing a newer approach to identifying group membership among actors and non-actors using a novel set of measures of creativity and other psychological dimensions. The authors presented a revised manuscript that addressed prior critiques meaningfully, all of which improved the manuscript thus far. Below are a few remaining areas to consider for revision to further strengthen the paper.

1. The organization about self-report measures is much improved from the prior version. It is much clearer how personality dimensions are being advanced with this study, and how grit and other creative activities would be useful. However, it would be beneficial to have a little more description about grit and creative activities to further justify their specific inclusion in the study, beyond their novelty.
2. Thank you for providing the frequency of participants by year in program. I agree with the author’s comment about university productions potentially providing more opportunities for involvement making their number of productions similar. Were there any questions about the roles the students and professionals had in each production? Perhaps a student was in many productions, but always part of the chorus instead of a lead. Any data about the depth of actors’ roles would be another great addition to the manuscript.
3. The addition about the natural development of volatility was a good point. However, I think it opens the possibility that other developmental patterns from adolescence to adulthood may be useful in distinguishing student vs professional actors, such as with the other-emotional appraisal construct. For example, typical brain development of the neurologic system responsible for seeking rewards is highly influenced by the rewards of peers, and develops earlier than the control system responsible for self-management which continues developing into the mid-20s. Thus, the college actors might be attuned to others’ emotions and more likely to react to them than their professional counterparts who have a fully developed control system that can ignore their emotional awareness as necessary. This example and other developmental patterns could be considered for their effect on identifying actor group membership. If interested in brain development of these systems and social implications, you might check some of Steinberg’s work on social neuroscience (e.g., 2008, 2010).

Minor revision

1. Table 2 concisely offers a substantial amount of information in a small space. However, the significant group differences are not always obvious. It might be worthwhile to denote which groups were significantly different (e.g., bold the M & SDs so you don’t have to add more physical text).
